# Exploring Suspected Diagnoses in Elderly Patients: A Case Study of Potential Necrotizing Otitis Externa

**DOI:** 10.7759/cureus.49801

**Published:** 2023-12-01

**Authors:** Luís A Rocha, Tiago Costa, Luciana Silva, Rafaela Veríssimo

**Affiliations:** 1 Internal Medicine, Vila Nova de Gaia/Espinho Hospital Centre, Vila Nova de Gaia, PRT; 2 Internal Medicine and Geriatrics, Vila Nova de Gaia/Espinho Hospital Centre, Vila Nova de Gaia, PRT

**Keywords:** diabetes mellitus in elderly, pseudomonas aeruginosa (p. aeruginosa), bone lesion, elderly patients, necrotising otitis externa

## Abstract

Necrotizing otitis externa (NOE) is a rare invasive infection affecting the EAC and the base of the skull. This condition is more prevalent in the elderly, diabetics, and immunocompromised individuals, often attributed to the bacterium *Pseudomonas aeruginosa*. In this case report, we present the clinical scenario of a 90-year-old woman with a history of diabetes and epilepsy. Initially admitted with acute pyelonephritis, fever, and prostration, she subsequently developed left facial paralysis during treatment. Cranial computed tomography (CT) revealed inflammation in the middle ear and bone erosion of the facial nerve canal. The examination by the Department of Ear, Nose, and Throat (ENT) describes that NOE would be the most likely diagnosis. Given the patient's comorbidities and the severity of the disease, the treatment was initiated empirically and later adjusted based on the culture and sensitivity results with ceftazidime. NOE is a critical condition requiring early diagnosis and interdisciplinary collaboration due to the associated risk of complications. Adequate glycemic control is imperative, and the judicious use of antibiotics is crucial in light of escalating resistance.

## Introduction

Necrotizing otitis externa (NOE), also known as malignant external otitis, is a severe infection associated with a high mortality rate. It originates in the external auditory canal (EAC) with the potential to extend to the base of the skull. The term "malignant" was introduced by Chandler in 1968, though the first case was reported by Toulmouche in 1838 [1]. Subsequently, Evans and Richards added the term "necrotizing," refining the clinical definition of the pathology. Despite the term "malignant," NOE is not a neoplastic disease, and the use of "otitis external" may be imprecise, as the infection can extend to deeper planes beyond the EAC [2]. The incidence is higher in individuals over 65 years old, with the elderly being more susceptible due to conditions compromising the immune system, such as diabetes mellitus (DM) and chronic kidney disease (CKD) [3]. *Pseudomonas aeruginosa *is the most prevalent pathogenic agent, although other bacteria, including Methicillin-resistant *Staphylococcus aureus* (MRSA), and fungal species can also cause the disease, specifically *Aspergillus* spp and *Candida* spp. Effective treatment requires early diagnosis, necessitating a high index of suspicion, especially in the early stages, which may resemble classical external otitis, leading to treatment delays [4]. Common complications include osteomyelitis, cranial nerve paralysis (especially facial nerve), meningitis, and cerebral abscess [5].

## Case presentation

A 90-year-old woman, partially dependent on activities of daily living, with a medical history of DM and epilepsy, was admitted to the internal medicine service for lethargy, fever, and vomiting. Initially diagnosed with acute pyelonephritis, she was empirically treated with sulfamethoxazole-trimethoprim. The patient's family reported a recent history of otorrhea and external otitis in the left ear, recently treated with oral amoxicillin and topical ofloxacin. There was no report of vertigo, recent acute hearing loss, diminished olfaction, oculomotor disturbance, or facial sensitivity and strength alterations.

On the sixth day of hospitalization, the patient exhibited an increase in inflammatory parameters, with C-reactive protein (CRP) rising from 6.2 mg/dL to 11.4 mg/dL and erythrocyte sedimentation rate (ESR) reaching 103 mm/h, along with a worsening of renal function with creatinine levels reaching 1.92 mg/dL, partially attributed to the infection and associated dehydration. Urine culture revealed the presence of *Escherichia coli*, susceptible to the ongoing antibiotic therapy. Renal function improved after fluid therapy, although the patient remained subfebrile. The patient's difficult glycemic control is also noteworthy.

**Figure 1:**
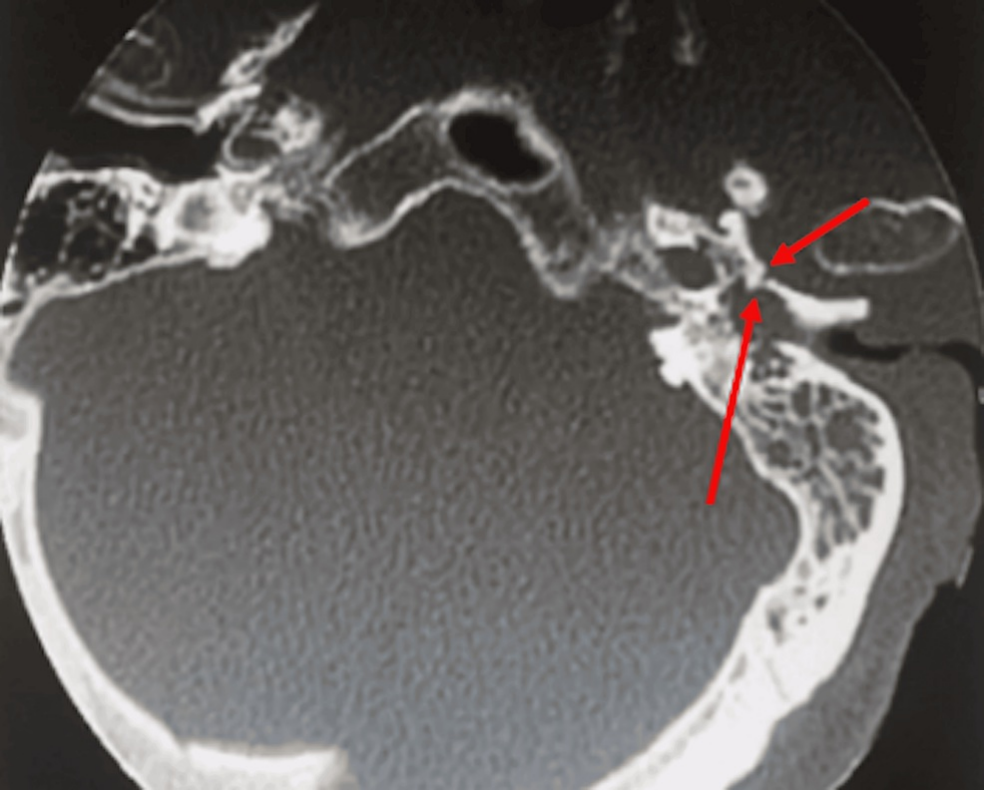
Cranial computed tomography Red arrows showing bone invasion of the mastoid cells.

On the tenth day, the patient developed left facial paralysis. Cranial computed tomography (CT) demonstrated an inflammatory/infectious process centered on the left middle ear, accompanied by erosion of the glenoid cavity wall and soft tissue components in the underlying masticatory and parapharyngeal spaces. Erosion of some caudal mastoid cells was also observed, as well as in the lower part of the mastoid segment of the facial nerve canal (Figure 1).

Chronic otitis media, carcinoma, and mucormycosis of the ear canal are considered. However, only microbiological culture was performed, and since *Pseudomonas* spp was isolated, fungal culture was not pursued. Neoplastic context was not excluded by biopsy, as the patient's prognosis was extremely poor, and it would not have altered the therapeutic approach. This decision was discussed with the ENT department, and the patient initiated empirical treatment with ceftriaxone until the cultural result of the EAC exudate revealed the presence of *P. aeruginosa*. After a discussion with the infection prevention and antibiotic resistance department, targeted antibiotic therapy with ceftazidime was initiated.

Throughout the hospitalization, the patient experienced pain, predominantly nocturnal, associated with erythema around the ipsilateral temporomandibular joint (TMJ) and exudate in the EAC. There was a compromise of the facial nerve with associated paralysis. Dysphagia was also observed, potentially indicating involvement of the glossopharyngeal, vagus, and hypoglossal nerves, albeit partially attributable to progressive prostration. 

Due to the patient's debility, diabetes, isolation of *Pseudomonas*, and the observed alterations in both physical examination and imaging (pain, swelling exudate, and granulation tissue in the EAC), the probability of NOE was high, and the diagnosis was presumed.

The debridement is the sole surgical procedure indicated. Nevertheless, in this case, and as discussed with the department ENT sice, we deemed its benefit to be lacking, considering the patient's low physiological reserve and the associated poor prognosis. The decision entailed targeted antibiotic therapy and symptomatic control.

The patient completed only two weeks of ceftazidime therapy and subsequently succumbed.

## Discussion

NOE is a severe and aggressive infection that primarily affects elderly individuals, particularly those with DM and other immunosuppressive conditions [2]. DM emerges as a predominant risk factor, being present in 90% to 100% of reported NOE cases. The underlying rationale for this association lies in the ability of DM to predispose patients to this infection. DM induces microangiopathy in the auditory canal, compromising the immune system's ability to combat the infection and rendering patients more susceptible to this process. Additionally, factors such as increased cerumen pH in diabetics and iatrogenic trauma (recent surgeries or the presence of hearing aids) are considered predisposing factors for NOE [6]. On the other hand, it has been observed that 27.8% of NOE cases have hypertension, which may contribute to microangiopathy [3]. The most common pathogen is *P. aeruginosa*, accounting for 50-90% of cases, followed by other pathogens such as *Proteus mirabilis*, *Aspergillus fumigatus*, *Proteus* spp., *Klebsiella* spp., and *Staphylococci* [7]. As of 2020, there was only one series of about 80 cases [8].

Although NOE is a rare condition, its severity and high mortality rate justify the need for early diagnosis and proper treatment. NOE poses a diagnostic dilemma, especially in elderly patients with comorbidities, and in medical settings where chronic diseases are prevalent. However, diagnosing it can be challenging since early symptoms, such as ear pain and purulent otorrhea, are similar to those of common external otitis, leading to treatment delays. The persistence of ear pain, along with the emergence of purulent otorrhea containing granulation tissue, and the presence of DM, or immunosuppression, are valuable indicators that can raise suspicion of NOE.

The diagnosis can only be confirmed if the following major criteria are met: pain, often disproportionate to physical examination, swelling, exudate, granulation tissue in the EAC, absence of improvement with local treatment for more than one week, microabscess (when surgical intervention is performed), and positive bone scintigraphy with Technetium-99m. We can only assert that the patient met the initial criteria, as surgical intervention and scintigraphy were not conducted. However, minor criteria were fulfilled, such as advanced age, associated debilitating condition, DM, and involvement of cranial nerves. For a diagnosis of NOE, all major criteria must be met [9]. Therefore, we cannot affirm that the definitive diagnosis was NOE but rather a strong suspicion.

Ninety percent of cases present otalgia as the predominant symptom, often of a lancinating nature and radiating to the TMJ, mainly manifesting at night [10]. The patient in this case experienced intense left-sided otalgia associated with ipsilateral facial paralysis. This otalgia can culminate in focal deficits due to the involvement of facial, hypoglossal, abducens, trigeminal, glossopharyngeal, vagus, and accessory spinal nerves [11]. Dysphagia with prostration was also observed in the presented case.

Surgical excision plays no role in current treatment, although it was mentioned and used before the availability of systemic antipseudomonal antibiotics. Debridement and biopsy are the only surgical procedures currently indicated. The lack of surgical intervention in the presented case aligns with the current treatment approach.

Regarding the role of hyperbaric oxygenotherapy, it has been used on occasion with mixed results and may be considered as an adjuvant treatment for refractory cases, although there is no evidence demonstrating the effectiveness of this treatment. The presented case did not involve hyperbaric oxygenotherapy, in line with the uncertainty and mixed outcomes associated with its use in NOE treatment [12].

Olfactory, oculomotor, and trochlear nerves, on the other hand, do not seem to be affected by this disease. Imaging studies play a crucial role in the diagnosis and assessment of infection extension. CT and magnetic resonance imaging (MRI) are essential to identify bone erosions and determine the infection's spread. CT is particularly effective in detecting bone changes, although it may not be as sensitive in the early stages of the disease [13]. Systemic antibiotics, such as fluoroquinolones, have demonstrated efficacy and reduced the need for hospitalization [14]. Regular debridement in NOE cases is rare, even when hospitalization is necessary, largely due to the effectiveness of anti-pseudomonal agents. The initial approach is weighed and depends on the severity of the disease, and the treatment duration of four to six weeks may be insufficient for patients at high risk of recurrent infections. However, the ideal duration of therapy for osteomyelitis in NOE remains uncertain [11].

## Conclusions

The in-depth analysis of this case underscores the pivotal role of understanding NOE in shaping its prognosis. The severity and intricacy of NOE necessitate meticulous consideration of factors such as diabetes control, inflammatory parameters, and imaging findings, all significantly contributing to the disease's progression. Insights from discussions spotlight specific risk factors, notably cranial involvement, serving as a key indicator of a less favorable prognosis. The challenges in long-term management are palpable, underscoring the need for continuous research and innovative approaches. The observed reduction in mortality rates signifies notable advancements in NOE diagnosis and treatment.

Furthermore, the ongoing discourse surrounding this case reaffirms the critical need for sustained research efforts, collaborative strategies, and personalized management approaches to augment our comprehension and refine therapeutic interventions for NOE. Effectively navigating the intricate interplay of factors influencing NOE prognosis demands the amalgamation of clinical insights and ongoing scientific progress, which remains paramount for optimizing patient care and outcomes in the context of this rare and severe otological condition.
